# The complete mitochondrial genome of the hybrid sturgeon of *Huso dauricus* (♀) × *Acipenser schrenckii* (♂)

**DOI:** 10.1080/23802359.2016.1275838

**Published:** 2017-01-11

**Authors:** Xueqing Liu, Hejun Du, Kan Xiao, Yacheng Hu, Juanjuan Liu, Xun Zhao

**Affiliations:** a Institute of Chinese Sturgeon, China Three Gorges Corporation, Yichang, China;; b Hubei Key Laboratory of Three Gorges Project for Conservation of Fishes, Institute of Chinese Sturgeon, China Three Gorges Corporation, Yichang, China

**Keywords:** *Huso dauricus*, *Acipenser schrenckii*, hybrid sturgeon, mitochondrial genome

## Abstract

The complete mitochondrial genome sequence of the hybrid of *Huso dauricus* (♀) × *Acipenser schrenckii* (♂) is described in this study. The 16,693bp long circular molecule consisted of 13 protein-coding genes, 2 rRNA genes, 22 tRNA genes, and a control region, showed a typical vertebrate pattern. All genes were encoded on the heavy strain except for ND6 and eight tRNA genes. Base composition of the heavy strain was A(30.40%), T(24.19%), C(29.26%), G(16.16%), and with A + T bias of 54.59%. Comparing with the complete mitochondrial genome of its parents, the hybrid sturgeon of *Huso dauricus* (♀) × *Acipenser schrenckii* (♂) was consistent with a maternal inheritance. The complete mitogenome of the hybrid sturgeon of *Huso dauricus* (♀) × *Acipenser schrenckii* (♂) provides an important dataset for the exploration of mitochondrial inheritance mechanism.

*Huso dauricus* and *Acipenser schrenckii* are two species of Acipenseriformes endemic to the Amur River basin in Russia and in China. They have great aquaculture potential due to their high economic values. However, growth depression, late sexual maturity, and disease susceptibility restrict the culture of population of *H. dauricus* and *A. schrenckii*. Because of heterosis, hybrid sturgeon has been an important species in sturgeon aquaculture in China (Sun et al. 2011). Recently, due to the damming of rivers, water pollution, and overfishing, natural resources of those two species have been assessed as Critically Endangered by IUCN (Ruban & Qiwei 2010).

In this study, we sequence the complete mitochondrial DNA sequence of the hybrid sturgeons of *H. dauricus* (♀) × *A. schrenckii* (♂) obtained by artificial hybridization from the Institute of Chinese Sturgeon (N 30°46′9.516″, E 111°18′51.084″), Yichang, China. This specimen was stored in 95% ethanol with accession number: 20160124BZ1. Total genomic DNA was extracted from caudal fins by a traditional phenol–chlorofrom method (Sambrook & Russell 2002). Eight pairs of primers were used to amplify the hybrid sturgeon of *H. dauricus* (♀) **×** *A. schrenckii* (♂) mitogenome according to the mitogenome of *A. schrenckii* (KC905169) and *H. dauricus* (KJ402277). The mitogenome data of the hybrid sturgeon of *H. dauricus* and *A. schrenckii* have been deposited in GenBank with the accession number: KY132098.

The complete mitogenome of the hybrid sturgeon of *H. dauricus* (♀) × *A. schrenckii* (♂) was a circular molecule with a length of 16,693 bp, including 13 protein-coding genes, 22 tRNA genes, 2 rRNAgenes, and 1 non-coding region (D-loop). This gene arrangement of the hybrid sturgeon of *H. dauricus* (♀) **×** *A. schrenckii* (♂) is similar to the typical vertebrate mitochondrial genomes (Saitoh et al. 2006). The overall base composition of the heavy strain was A(30.40%), T(24.19%), C(29.26%), G(16.16%), and the content of A + T in the complete genome was 54.59%. Most of the genes were encoded on the heavy strand except ND6 and 8 tRNA [tRNA–Gln, **–**Ala, **–**ASn, **–**Cys, **–**Tyr, **–**Glu, **–**Pro, and **–**Ser (TGA)].

As showed in neighbour-joining tree (by MEGA6.0, Arizona State University, America) based on mitochondrial 13 protein genes nucleotide sequences of the hybrid and other 12 kinds of sturgeons was constructed with 1000 bootstrap replicates (Figure 1). Our sequence was phylogenetically clustered with *H. dauricus*, which is consistent with the mitochondrial inheritance mechanism. The complete mitochondrial genome sequence of the hybrid sturgeon of *H. dauricus* (♀) × *A. schrenckii* (♂) provides an important data set for further study in mitochondrial inheritance mechanism.

**Figure 1.**
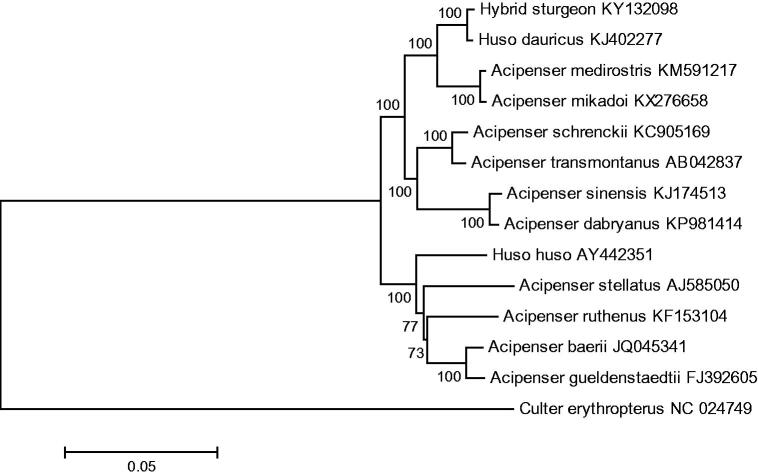
The consensus phylogenetic relationship of the hybrid sturgeon of *Huso dauricus* (♀) × *Acipenser schrenckii* (♂) with other sturgeons. The numbers along the branches are Bayesian posterior probability and bootstrap values for NJ, estimated for concatenated mitochondrial protein sequences.
